# Predicting environmentally suitable areas for *Anopheles superpictus* Grassi (*s.l.*), *Anopheles maculipennis* Meigen (*s.l*.) and *Anopheles sacharovi* Favre (Diptera: Culicidae) in Iran

**DOI:** 10.1186/s13071-018-2973-7

**Published:** 2018-07-03

**Authors:** Ahmad Ali Hanafi-Bojd, Mohammad Mehdi Sedaghat, Hassan Vatandoost, Shahyad Azari-Hamidian, Kamran Pakdad

**Affiliations:** 1 Department of Medical Entomology & Vector Control, School of Public Health, Tehran University of Medical Sciences, Tehran, Iran; 2 Department of Environmental Chemical Pollutants and Pesticides, Institute for Environmental Research, Tehran University of Medical Sciences, Tehran, Iran; 3 Research Center of Health and Environment, School of Health, Guilan University of Medical Sciences, Rasht, Iran; 4 Department of Parasitology and Mycology, Paramedical School, Shahid Beheshti University of Medical Sciences, Tehran, Iran

**Keywords:** *Anopheles superpictus* (*s.l*.), *An. maculipennis* (*s.l*.), *An. sacharovi*, Ecology, Spatial distribution, Modeling, Iran

## Abstract

**Background:**

Malaria is an important mosquito-borne disease, transmitted to humans by *Anopheles* mosquitoes. The aim of this study was to gather all records of three main malaria vectors in Iran during the last decades, and to predict the current distribution and the environmental suitability for these species across the country.

**Methods:**

All published documents on *An. superpictus* Grassi (*s.l*.), *An. maculipennis* Meigen (*s.l.*) and *An. sacharovi* Favre during 1970–2016 in Iran were obtained from different online data bases and academic libraries. A database was created in ArcMap 10.3. Ecology of these species was analyzed and the ecological niches were predicted using MaxEnt model.

**Results:**

*Anopheles superpictus* (*s.l*.) is the most widespread malaria vector in Iran, and exists in both malaria endemic and non-endemic areas. Whereas *An. maculipennis* (*s.l*.) is reported from the northern and northwestern parts, *Anopheles sacharovi* is mostly found in the northwestern Iran, although there are some reports of this species in the western, southwestern and eastern parts. The area under receiver operating characteristic (ROC) curve (AUC) for training and testing data was calculated as 0.869 and 0.828, 0.939 and 0.915, and 0.921 and 0.979, for *An. superpictus* (*s.l*.), *An. maculipennis* (*s.l*.) and *An. sacharovi*, respectively. Jackknife test showed the environmental variable with highest gain in the predicting power of the model when used in isolation was annual precipitation for *An. superpictus* (*s.l*.) and *An. maculipennis* (*s.l*.), and precipitation of the driest quarter for *An. sacharovi*.

**Conclusions:**

Despite this range, global warming may increase the potential risk for malaria transmission in some cleared-up areas, where these proven vectors are active. Mapping and prediction of spatial/temporal distribution of these vectors will be beneficial for decision makers to be aware of malaria transmission risk, especially in the western parts of the country.

**Electronic supplementary material:**

The online version of this article (10.1186/s13071-018-2973-7) contains supplementary material, which is available to authorized users.

## Background

The incidence of malaria has been steadily decreasing in Iran from 16,747 to 632 during 2001–2015, with the majority of cases imported. Iran is now focused on the elimination of malaria, and the tools implemented are vector control based on long-lasting insecticide-treated nets (LLINs) and indoor residual spraying (IRS), as well as improved diagnosis and free of charge access to drugs *via* the health system. There is a heavy focus of vector control in the southeastern region of Iran because local transmission occurs in this area, noting that insecticide resistance to pyrethroids has been recently detected in this area [1–4]. Throughout Iran seven anopheline species are primary or secondary vectors of malaria: *An. culicifacies* Giles (*s.l*.), *An. dthali* Patton, *An. fluviatilis* James (*s.l*.), *An. maculipennis* Meigen (*s.l*.), *An. sacharovi* Favre, *An. stephensi* Liston and *An. superpictus* Grassi (*s.l*.) [5]. The relative importance of these vector species varies across the country, with *An. stephensi*, *An. culicifacies* (*s.l*.) and *An. fluviatilis* (*s.l*.) being relevant in southern Iran [6], and *An. superpictus* (*s.l*.), *An. maculipennis* (*s.l*.) and *An. sacharovi* being relevant across the central, northern and northwestern Iran [5]. It is noteworthy that *An. sacharovi* was known important vector during epidemics occurred in northwestern Iran two decades ago [7].

*Anopheles superpictus* (*s.l*.) is a Palaearctic species recorded from Afghanistan, Albania, Algeria, Armenia, Azerbaijan, Bosnia and Herzegovina, Bulgaria, Croatia, Cyprus, Egypt and France. In addition, the species is also present in Georgia, Greece, Iran, Iraq, Israel (and Gaza Strip & West Bank), Italy, Jordan, Kazakhstan, Kyrgyzstan, Lebanon, Libya, Macedonia, Pakistan, Russia, Spain, Tajikistan, Tunisia, Turkey, Turkmenistan, Uzbekistan and Yugoslavia [5, 8–13]. *Anopheles superpictus* (*s.l*.) has the most extensive geographical distribution in Iran [14–18]. This species was identified as the main vector of malaria in the central plateau of Iran, but a secondary vector in southern areas [5, 18]. Previous studies in the country found three genotypes of this species based on cytochrome *c* oxidase 1 (*cox*1) data, although there is no information concerning its distribution ranges in other countries [19]. Harbach [20] listed two species, A and B, in the complex according to internal transcribed spacer 2 (ITS2) data. Studies in Iran and other countries where this mosquito is distributed reported zoophilicity feeding behavior, but it can only feed on human in the absence of other animal baits. Human blood index of this species in Iran is reported as 11.4% [9, 21]. Under laboratory conditions, artificially infected *An. superpictus* (*s.l*.) has completed sporogonic stages of *Plasmodium vivax* within 11.7 days [22]. This species was found to be naturally infected by sporozoite in Iran with an infection rate of 0.65–4.7% [23]. Recent study showed a decrease in *An. superpictus* (*s.l*.) distribution within western Zagros region and an increasing trend in distribution in the southern boundary [24].

The *Anopheles maculipennis* Group includes seven species in Iran. There are no reliable morphological characters for distinguishing all species of this group. The most common characters to identify members of this group are egg pattern, polytene chromosome, isoenzymes and species-specific polymerase chain reaction (PCR) [25]. Indeed, in the keys, only *An. sacharovi* is separated from other species in Iran [26]. With a human blood index of about 5% and reported sporozoite rate of 0.33%, *An. sacharovi* had an important role in malaria transmission in the Caspian Sea littoral in past decades [21]. Recent studies reported this *Anopheles* species from Iran, especially in the northern and western parts [14, 16, 27–33]. Global warming in the past 40 years has led to changes in spatial distribution of this species group; thus, the distribution area of *An. maculipennis* (*s.s.*) is expanding at an average speed of about 30 km per year; between 2008–2009 [34].

*Anopheles sacharovi* is reported from coastal parts of Italy, Sardinia, Corsica, Croatia, Republic of Macedonia, Albania, Bulgaria, Romania, southern regions of the former USSR, Turkey, Lebanon, Israel, Jordan, Syria, Iraq and Iran [35]. It is the most important malaria vector in Turkey, northwest border of Iran [36]. Twenty years ago, an epidemy of malaria occurred in northwestern Iran, where *An. sacharovi* was identified as the vector [7]. This species is distributed from northwest to southeast of Iran, alongside the Zagros Mountains [5, 37]. A human blood index of 38.5% shows the high tendency of this *Anopheles* species to human hosts [7]. This species has a good potential for malaria transmission to humans, where the infected host is available.

Mapping the potential distribution of malaria vectors in Iran is an important issue, because the country is in the phase of malaria elimination. This approach has been considered by different researchers across malaria endemic/at risk areas in the world, although their methodologies were different [6, 38–40]. Modeling by means of climatic and environmental variables will help us to predict the current and future spatial distribution of targeted species. Ecological niche modeling using MaxEnt model [41] is a commonly used method for predicting the potential presence of different species including malaria vectors [6, 38]. MaxEnt uses only presence data. This is appropriate for species distribution modelling concerned with predicting areas of potential species occurrence, recognizing that absence data are rarely available or reliable. Furthermore, the predictive performance of MaxEnt is consistently competitive with the highest performing methods [41–43].

The aim of this study was to gather all records of three malaria vectors, i.e. *Anopheles superpictus* (*s.l*.), *An. maculipennis* (*s.l*.) and *An. sacharovi*, in Iran during the last decades, and to predict the current distribution and environmental suitability of these species across the country.

## Methods

### Study area

**Fig. 1:**
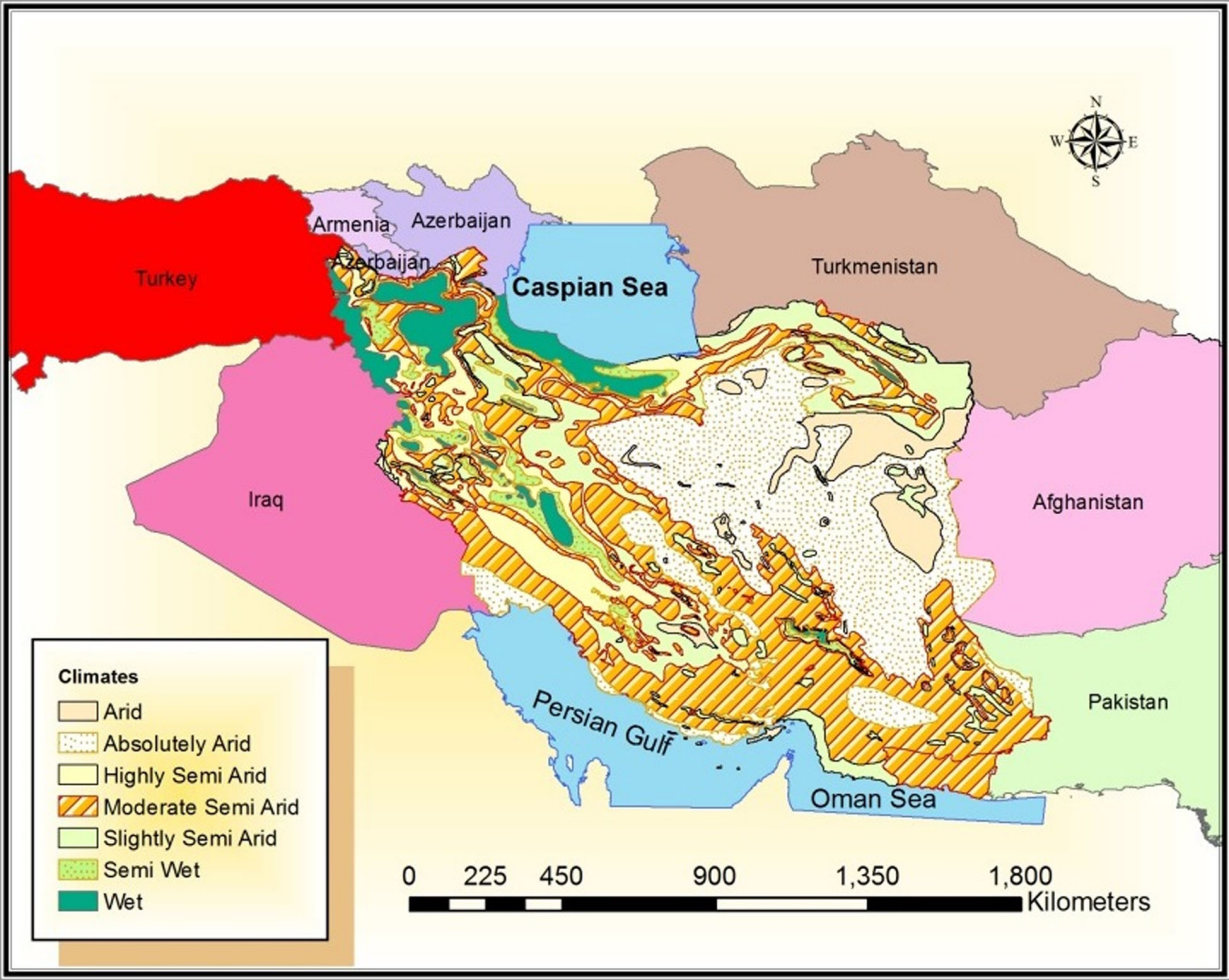
Iran climate map (Koppen climate classification) and its neighboring countries

Iran with an area of 1,648,000 km^2^ is located in Middle East and bordered by Armenia, Azerbaijan, Caspian Sea and Turkmenistan in the north, Turkey and Iraq in the west, Persian Gulf and Oman Sea in the south, and Afghanistan and Pakistan in the east. Two main mountainous ridges in the north (Alborz) and the west (Zagros) affect the climate of Iran. Thus, seven main climates exist, i.e. arid, highly arid, absolutely arid, moderate semi-arid, slightly semi-arid, semi wet and wet (Koppen climate classification). Most areas in the country are affected and covered by three climates: arid, absolutely arid and moderate semi-arid (Fig. 1). These natural conditions and topography provide different ecological niches suitable for various plants and animals species, including mosquitoes.

### Species data collection

In this study, various scientific databases were searched (Google Scholar, PubMed, SID, Ovid Medline, Web of Science, Irandoc and Magiran) using the scientific names of the targeted species, i.e. “*An. superpictus*”, “*An. maculipennis*” and “*An. sacharovi*”. All documents that were published during 1970–2016 were considered and reviewed. To avoid false data, one of the main inclusion criteria was to enter information which published by experts in the field of mosquito taxonomy. According to the reports, distribution data of target species were reported in different levels including village, city, county and province. Although we included the spatial distribution of these species at the county and province level, just the reported x and y coordinates were used for modeling. Also, if a study reported a mosquito species at village level, we extracted the geographical coordinates of the village from the database of Iranian villages and used for mapping and modeling. All occurrence coordinates that used for mapping and modeling were included in Additional file 1.

### Environmental and bioclimatic data

Species distribution models (SDMs), also known as bioclimatic envelope models, are the most widely used approach for predicting suitable habitats for different species. Bioclimatic variables are derived from the monthly temperature and rainfall values in order to generate more biologically meaningful variables. The bioclimatic variables represent annual trends, seasonality and extreme or limiting environmental factors. In this study, the bioclimatic data were downloaded from the WorldClim database. This dataset is available as individual rasters spanning the inhabited continents, presented as latitude/longitude coordinates in WGS84. All 19 bioclimatic variables with a spatial resolution of 1 km^2^ were downloaded for the period of 1950–2000 and clipped using shapefile of Iran border as analysis mask (version1.4, http://www.worldclim.org/bioclim). Band collection statistics analysis in ArcMap that provides *statistics* for the multivariate analysis of a set of raster was used to find bioclimatic variables with less than 0.8 correlations. So, the included variables in the model were: Bio1, annual mean temperature (°C); Bio6, minimum temperature of coldest month (°C); Bio11, mean temperature of coldest quarter (°C); Bio12, annual precipitation (mm); Bio14, precipitation of the driest month (mm); Bio17, precipitation of the driest quarter (mm); Bio18, precipitation of warmest quarter (mm). Two environmental variables, altitude (m) and Normalized Difference Vegetation Index (NDVI), were also used for modeling. Altitude was derived from Digital Elevation Model (DEM) of Iran, whereas NDVI was acquired from June 2016 image of MODIS satellite. To run MaxEnt model it is required to all environmental raster layers have identical cell sizes and projections, so these two environmental layers were rescaled and re-projected in ArcMap 10.3 to match WorldClim layer resolution (1 km^2^) and projection (WGS 1984).

### Ecological niche modeling

All selected environmental and bioclimatic variables for modeling, were converted to ASCII format for modeling in the next step by Maximum Entropy (MaxEnt) model ver 3.3.3 [41, 42]. MaxEnt takes a list of species presence locations as input, often called presence-only data, as well as a set of environmental predictors such as precipitation, temperature, vegetation, etc. across a user-defined landscape that is divided into grid cells. From this landscape, MaxEnt extracts a sample of background locations that it contrasts against the presence locations. Presence is unknown at background locations [44]. Here the default MaxEnt selection of background points was kept.

The contribution of the environmental and bioclimatic variables was tested by Jackknife analysis in MaxEnt model to get alternate estimates of which variables are most important in the model [45]. All variables with no contribution (0 values) in the test were excluded from the final analysis. In MaxEnt, presences can be split into ‘training’ and ‘test’ data. Training data are used to create the predictive model; test data are used to assess model accuracy. In our study, 80% of the occurrence records was randomly selected by the model and used as training data. The remaining 20% were used as test data. Then the model output was two AUCs for training and test data, as we presented in the results.

### GIS-based analysis and mapping data

Data records were mapped using ArcMap 10.3. Distribution points of *Anopheles superpictus* (*s.l*.), *An. maculipennis* (*s.l*.) and *An. sacharovi* in villages, where coordinates existed, and crossed with altitude and climate layers of Iran to find their distribution in different climates and altitudes. Prediction maps of environmental suitability for these species, produced by MaxEnt model, was then classified in ArcMap to obtain the area with a suitability of more than 60%. Then the main climatic variables including ranges, average annual temperature ± standard deviation, relative humidity and altitude were determined for this range.

## Results

### Spatial distribution

**Fig. 2:**
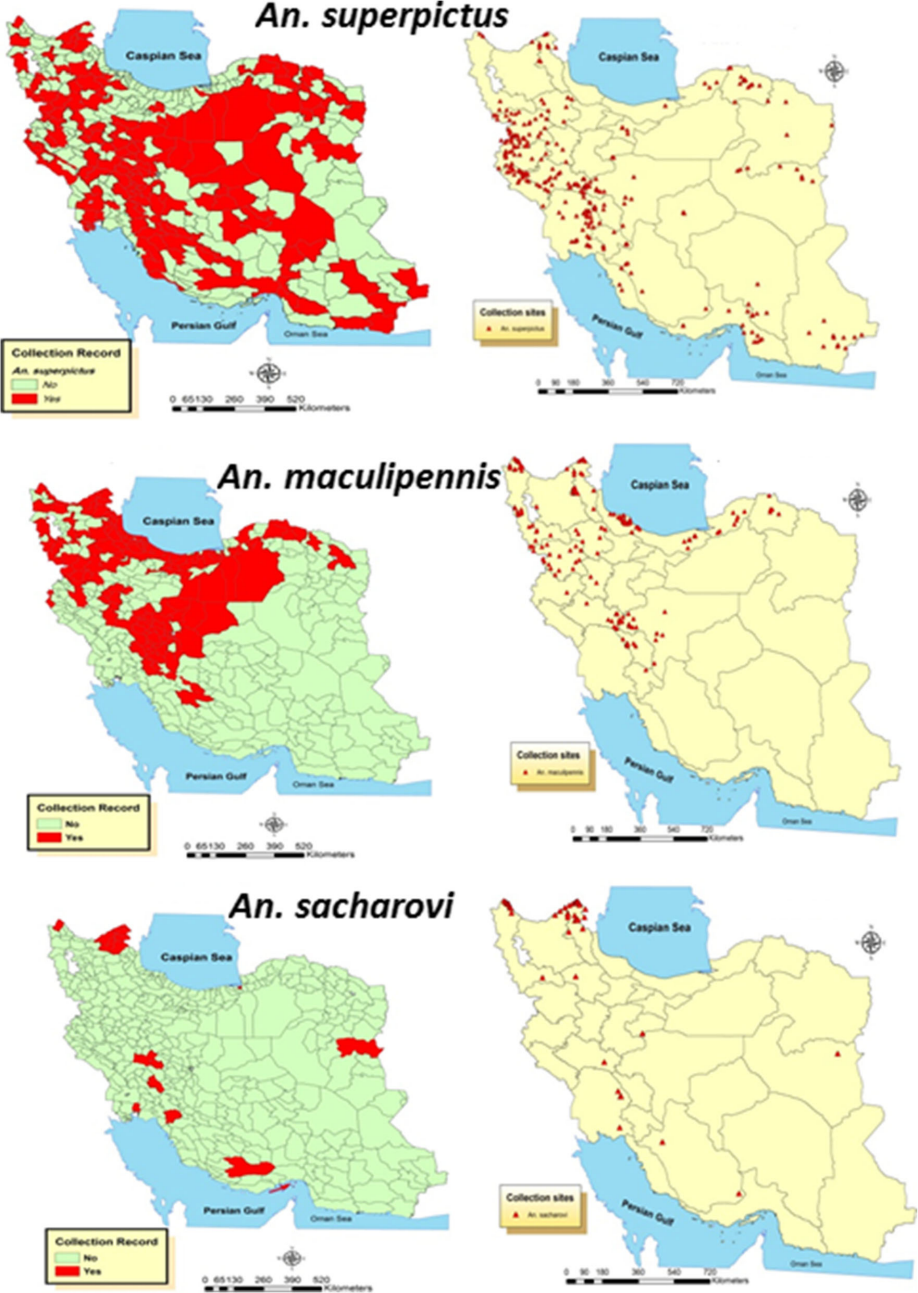
Spatial distribution of *Anopheles superpictus* (*s.l*.), *An. maculipennis* (*s.l*.) and *An. sacharovi* in Iran at the county (left) and village scales (right), 1970–2016.

Of over 200 records, 159 documents had information on the fauna of Culicidae in Iran. These documents were obtained, reviewed and their results recorded in the database. Of this number, there were 72 master and PhD theses, 52 articles in English, 25 Persian language papers, 8 abstracts and two research projects. Among the 159 reviewed documents, 67, 45 and 27 had reported spatial distribution of *An. superpictus* (*s.l*.), *An. maculipennis* (*s.l*.) and *An. sacharovi*, respectively. In this study we considered the distribution of these species in three scales: province, county and village or city level (as points of collection). With this approach *An. superpictus* (*s.l*.) was found in 29 out of 31 provinces and 152 counties in Iran during the last five decades (Table 1). During this period this species was collected from 296 collection sites. *Anopheles maculipennis* (*s.l*.) was reported from 20 provinces, 122 counties and 163 collection sites in Iran during the study period. Finally, *An. sacharovi* was recorded in 18 provinces, 22 counties (Table 1) and 50 collection sites (Fig. 2).

**Table 1:** Distribution of collection sites (in %) for the studied species in different climates of Iran, 1970–2016

Species	Moderate semi-arid	Highly semi-arid	Slightly semi-arid	Wet	Semi-wet	Arid	Absolutly arid
*An. superpictus* (*s.l*.)	33	28	13	9	13	1	3
*An. maculipennis* (*s.l*.)	22.3	26.5	6.8	30.2	14.2	0	0
*An. sacharovi*	64.3	14.3	14.3	0	4.7	2.4	0

Analysis of the three environmental variables in all recorded points of these malaria vectors in Iran showed that *An. superpictus* (*s.l*.) existed in an altitudinal range of 0–2716 m (mean ± standard deviation: 1124.80 ± 611.57 m) with annual mean temperature of 9.1–27.5 °C (16.7 ± 4.8 °C) and annual precipitation range of 54–940 mm (359 ± 169 mm). *Anopheles maculipennis* (*s.l*.) was also found in altitudinal range of -27–3483 m (1175 ± 752 m), annual mean temperature of 5.4–17.6 °C (13 ± 2.4 °C), and annual precipitation of 97–1496 mm (481 ± 309 mm). Finally, analysis of the environmental variables showed that *An. sacharovi* was found within an altitudinal range of 23–2113 m (7.9 ± 572 m), annual mean temperature of 8.8–25.4 °C (13.6 ± 3.4 °C), and annual precipitation range of 130–532 mm (349 ± 88 mm) in Iran.

Considering the climatic condition in the recorded points for these *Anopheles* species, *An. superpictus* (*s.l*.) was mostly found in moderate semi-arid (33%) and highly semi-arid (28%) climates whereas more than 44% of the occurrence records of *An. maculipennis* (*s.l*.) were in wet and semi-wet climates. However, no records of these species were reported from arid and absolutely arid climates. Also *An. sacharovi* collected mainly in moderate semi-arid climate (64.3%), while no record of its existance was found in wet and absolutly arid climates (Table 2).

**Table 2:** Distribution of three malaria vectors at the province and county levels in Iran

Species	Province	County	Collection sites
*An. superpictus* (*s.l*.)	Ardabil, Azarbaijan-e Gharbi, Azarbaijan-e Sharghi, Bushehr, Charmahal va Bakhtiari, Fars, Guilan, Golestan, Hamedan, Hormozgan, Ilam, Esfahan, Kerman, Kermanshah, Khorassan-e Razavi, Khorassan-e Jonoobi, Khorassan-e Shomali, Khuzestan, Kohgiluyeh va Buyerahmad, Kordestan, Lorestan, Markazi, Mazandaran, Qom, Semnan, Sistan va Baluchestan, Tehran, Yazd Zanjan	Parsabad, Meshginshahr, Germi, Fereydunshahr, Kalibar, Maku, Sanandaj, Divandarreh, Saqez, Baneh, Aligudarz, Qom, Gachsaran, Izeh, Bandar-e Mahshahr, Shiraz, Marvdasht, Larestan, Qaenat, Rudbar, Fuman, Khalkhal, Aran va Bidgol, Ardestan, Chadegan, Khomeynishahr, Semirom, Shahreza, Golpayegan, Mobarakeh, Naeen, Natanz, Najafabad, Shahinshahr va Meymeh, Tiran va Karvan, Faridan, Shemiranat, Zanjan, Abhar, Mahneshan, Khodabandeh, Tarom, Semnan, Damghan, Garmsar, Shahrud, Hamedan, Tuyserkan, Malayer, Nahavand, Tabriz, Ahar, Miyaneh, Hashtrud, Orumiyeh, Khoy, Mahabad, Miyandoab, Sardasht, Sahrekord, Ardal, Borujen, Farsan, Lordegan, Kuhrang, Kiyar, Mashhad, Kohgiluyeh, Quchan, Bijar, Sarvabad, Qorveh, Kamyaran, Marivan, Boyerahmad, Kalaleh, Neka, Mahallat, Khomeyn, Bojnurd, Shirvan, Maneh va Semelqan, Kermanshah, Tehran, Chah bahar, Saravan, Dayyer, Kangan, Dashtestan, Dashti, Tangestan, Varamin, Asadabad, Yazd, Ardakan, Bafq, Taft, Tabas, Mehriz, Gonabad, Neyshabur, Ilam, Torbat-e Jam, Torbat-e Heydariyeh, Daregaz, Sabzevar, Kashmar, Ahvaz, Abadan, Andimeshk, Dezful, Ramhormoz, Shadegan, Shushtar, Iranshahr, Konarak, Sarbaz, Abadeh, Sepidan, Arsanjan, Estahban, Darab, Fasa, Kazerun, Mamasani, Firuzabad, Khorramabad, Arak, Tafresh, Saveh, Shazand, Delijan, Bandarabbas, Minab, Bashagerd, Rudan, Eyvan, Darrehshahr, Dehloran, Mehran, Esfarayen, Ferdows, Eslamabad-e Gharb, Paveh, Qasreshirin, Jiroft, Rafsanjan, Shahrebabak, Kahnuj, Kerman, Bam, Zarand	296
*An. maculipennis* (*s.l*.)	Ardabil, Azarbayjan-e-Qarbi, Azarbayjan-e-Sharqi, Chaharmahal va Bakhtiari, Guilan, Golestan, Hamedan, Isfahan, Kermanshah, Khorassan-e-Razavi, Khorassan-e-Shomali, Kohgiluye va Boyerahmad, Kurdistan, Lorestan, Markazi, Mazandaran, Qazvin, Semnan, Tehran, Zanjan	Rasht, Astaneh-ye Ashrafiyeh, Astara, Amlash, Bandar-e Anzali, Rudbar, Rudsar, Shaft, Sumehsara, Fuman, Langerud, Lahijan, Masal, Siyahkal, Rezvanshahr, Tavalesh, Ardebil, Bilehsowar, Parsabad, Khalkhal, Meshginshahr, Namin, Nir, Germi, Kowsar, Esfahan, Aran va Bidgol, Ardestan, Chadegan, Khomeynishahr, Khansar, Semirom, Shahreza, Fereydunshahr, Falavarjan, Kashan, Golpayegan, Mobarakeh, Naeen, Natanz, Najafabad, Shahinshahr va Meymeh, Tiran va Karvan, Faridan, Lenjan, Damavand, Karaj, Shemiranat, Zanjan, Abhar, Mahneshan, Khodabandeh, Tarom, Semnan, Damghan, Garmsar, Shahrud, Hamedan, Tuyserkan, Malayer, Nahavand, Tabriz, Ahar, Kalibar, Maragheh, Marand, Miyaneh, Hashtrud, Orumiyeh, Poldasht, Piranshahr, Khoy, Maku, Mahabad, Miyandoab, Naqadeh, Sardasht, Sahrekord, Ardal, Borujen, Farsan, Lordegan, Kuhrang, Kiyar, Mashhad, Kohgiluyeh, Quchan, Sanandaj, Bijar, Divandarreh, Sarvabad, Saqez, Qorveh, Kamyaran, Marivan, Baneh, Dehgolan, Boyerahmad, Gorgan, Azadshahr, Bandar-e Gaz, Kalaleh, Minudasht, Aligudarz, Sari, Amol, Babol, Behshahr, Qaemshahr, Neka, Nowshahr, Tonekabon, Ramsar, Nur, Savadkuh, Mahallat, Khomeyn, Bojnurd, Shirvan, Maneh va Semelqan, Kermanshah, Tehran	163
*An. sacharovi*	Ardabil, Azarbayjan-e-Qarbi, Azarbayjan-e-Sharqi, Fars, Guilan, Golestan, Hormozgan, Isfahan, Kermanshah, Khorassan-e-Jonubi, Khuzestan, Kohgiluye va Boyerahmad, Kurdistan, Lorestan, Mazandaran, Qazvin, Qom, Zanjan	Bilehsowar, Parsabad, Meshginshahr, Germi, Fereydunshahr, Kalibar, Maku, Sanandaj, Divandarreh, Saqez, Baneh, Bandar-e Gaz, Aligudarz, Qom, Gachsaran, Izeh, Bandar-e Mahshahr, Shiraz, Marvdasht, Larestan, Qeshm, Qaenat	50

### Ecological niche modeling

#### *Anopheles superpictus* (*s.l*.)

**Fig. 3:**
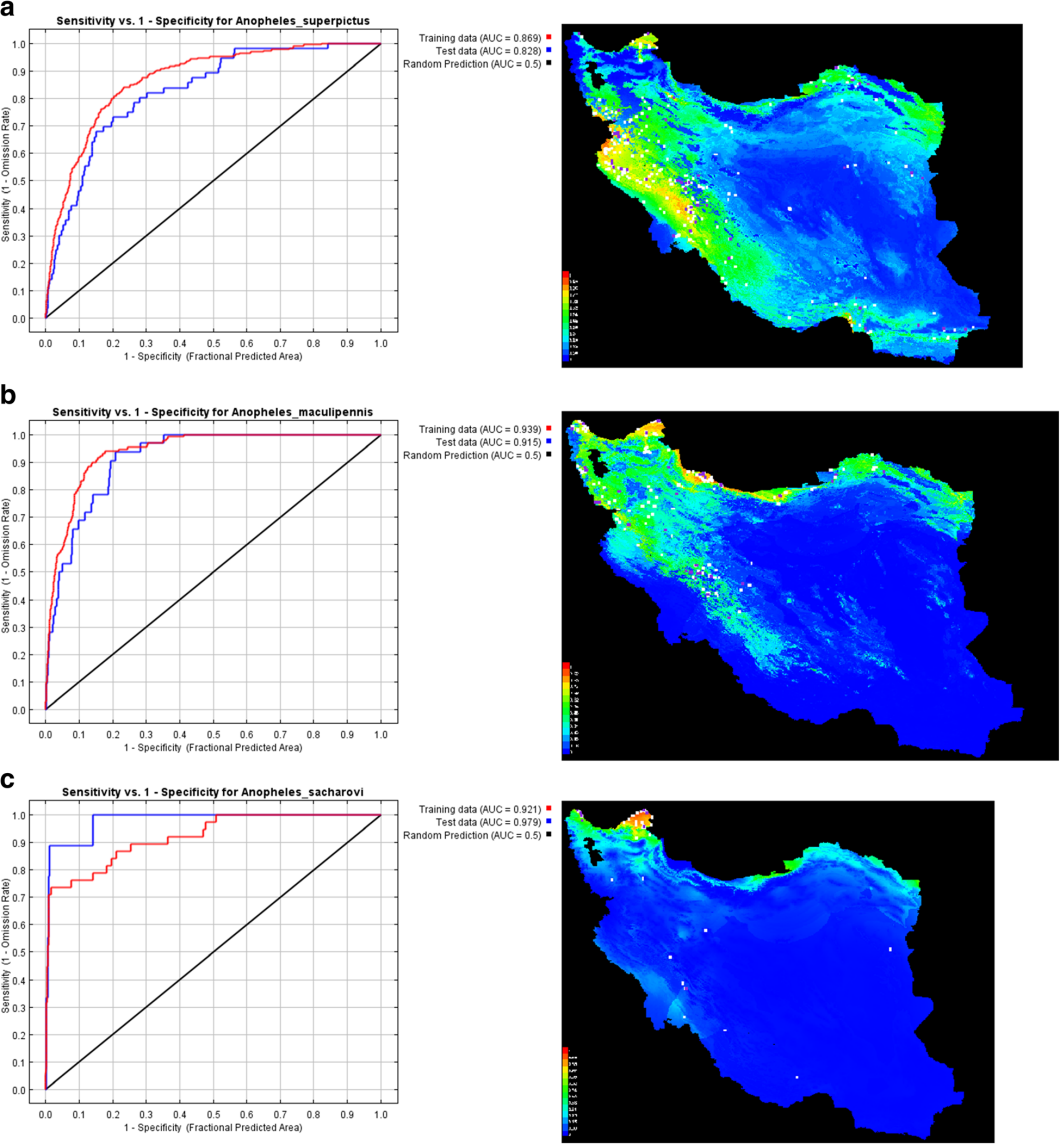
Representation of MaxEnt model (left) and receiver operating characteristic (ROC) curve (right) for three malaria vectors in Iran: *Anopheles superpictus* (*s.l*.) (**a**), *Anopheles maculipennis* (*s.l*.) (**b**) and Anopheles sacharovi (**c**)

As shown in the model output, the environmental suitability for *An. superpictus* (*s.l*.) was predicted to be present in most western parts of Iran (Fig. 3a), although there are some areas of high environmental suitability in northeastern, southwestern and southern regions as well. Warmer colors (red) show areas with higher environmental suitability for *An. superpictus* (*s.l*.); white dots show the presence locations used for training, while violet dots show test locations. The area under receiver operating characteristic (ROC) curve (AUC) for training and testing data was calculated as 0.869 and 0.828, respectively.

**Fig. 4:**
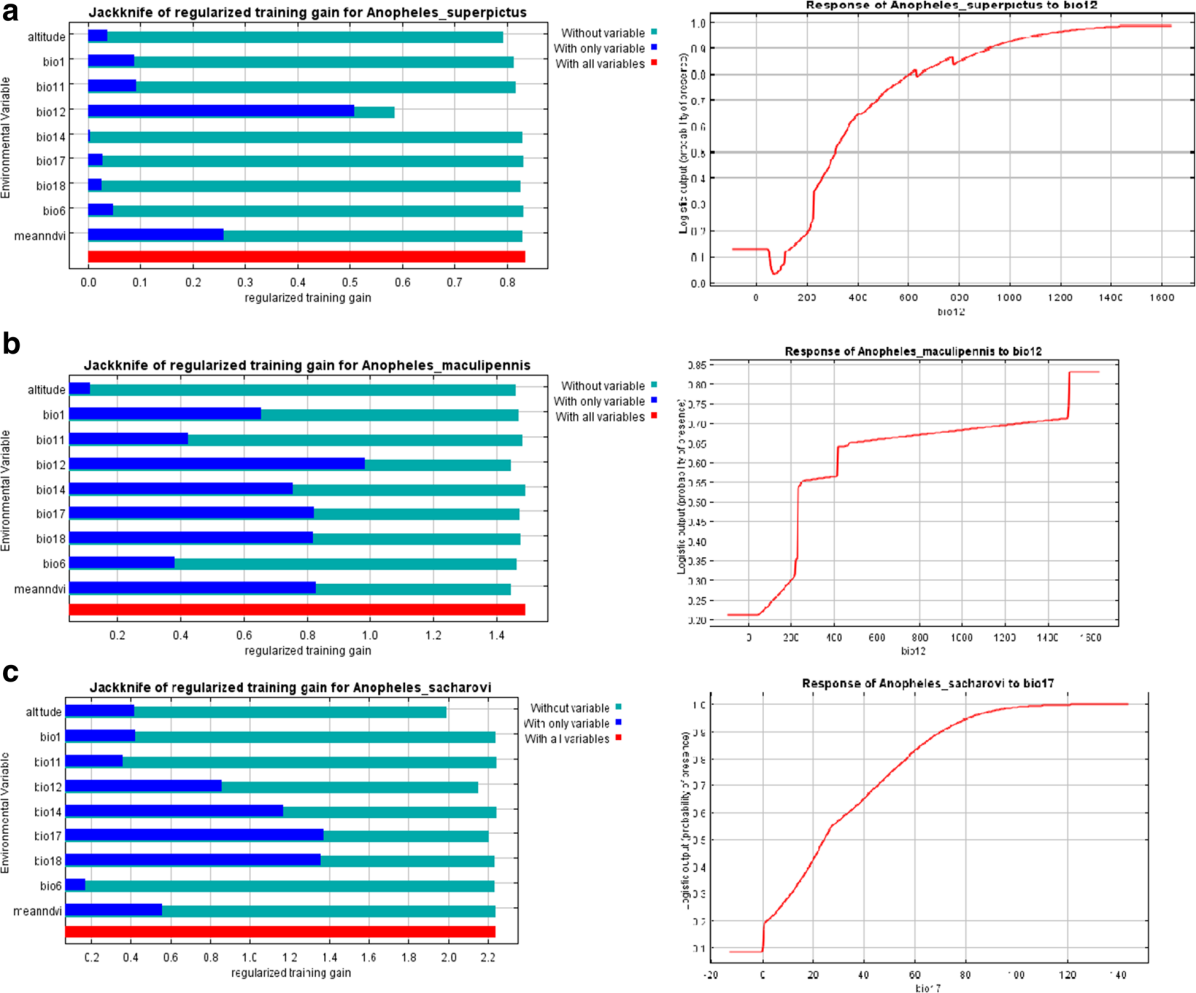
Result of the jackknife test of variable importance (left) and the curve of the most important variable in the model (right) for three malaria vectors in Iran. **a** *Anopheles superpictus* (*s.l*.). **b** *Anopheles maculipennis* (*s.l*.). **c** *Anopheles sacharovi*

Table 3 shows the contributed ratio of Bio 12 was more than other variables used in the model. The results of the jackknife test of variables’ importance indicated the environmental variable with highest gain in the predicting power of the model when used in isolation was Bio12 (annual precipitation). It appeared to have the most useful information by itself for predicting environmental suitability of *An. superpictus* (*s.l.*) It has been shown to decrease the gain in the predicting power of the model most when omitted. Environmental suitability for this *Anopheles* was found to be more with increasing annual rainfall (Fig. 4a).

**Table 3:** Analysis of variable contribution (%) for modeling three malaria vectors in Iran, shows which variables matter most for the species being modeled

Variable	*An. maculipennis* (*s.l*.)	*An. sacharovi*	*An. superpictus* (*s.l*.)
Altitude	5.7	20.1	3.9
Bio 1	3.3	0.1	22
Bio 6	1.1	0.6	4
Bio 11	10.9	6.5	2.3
Bio 12	66.7	12.7	31.3
Bio 14	2.1	0.1	2.2
Bio 17	0.6	58.5	2.9
Bio 18	8	0.7	16
NDVI	1.6	0.7	15.4

#### *Anopheles maculipennis* (*s.l*.)

As shown by the model output again, environmental suitability for *An. maculipennis* (*s.l*.) was predicted to be greater in north and northwestern parts of Iran (Fig. 3b), although there are some areas of high environmental suitability in northeastern part as well. Warmer colors (red) show areas with higher environmental suitability for *An. maculipennis* (*s.l*.); white dots show the presence locations used for training, while violet dots show test locations. AUC for training and testing data was calculated as 0.939 and 0.915, respectively.

Table 3 shows that the contribution ratio of Bio 12 was more than other variables used in modeling of this species. The results of the jackknife test of variable importance showed the environmental variable with the highest gain in the predicting power of the model when used in isolation was Bio12 (annual precipitation), which appeared to have the most useful information by itself, while NDVI showed the most decreased gain in the predicting power of the model when omitted. This showed that environmental suitability for *A. maculipennis* was found to be improved with increasing annual precipitation (Fig. 4b). On the other hand, more water availability would also increase the probability of presence.

#### *Anopheles sacharovi*

As the model output shows, the environmental suitability for *An. sacharovi* was predicted to be highest in north and northwestern parts of Iran (Fig. 3c), although there are some areas of high environmental suitability in northeastern region as well. Warmer colors (red) show areas with higher environmental suitability for *An. sacharovi*; white dots show the presence locations used for training, while violet dots show test locations. AUC for training and testing data was also calculated as 0.921 and 0.979, respectively.

Table 3 shows the contribution ratio for the different variables used in the model, i.e. which variables matter most for the species being modeled. As shown in the table, Bio 17 had the most contribution in modeling this species. The results of the jackknife test of variable importance showed that the environmental variable with highest gain in the predicting power of the model when used in isolation was Bio17 (Precipitation of the driest quarter). This appeared to have the most useful information by itself in predicting suitable environments for this malaria vector, but altitude has been shown to decreased gain in the predicting power of the model most when omitted. Environmental suitability for *A. sacharovi* was found to be more abundant with increasing rainfall in the driest quarter (Fig. 4c). In other words, species distribution for this *Anopheles* is influenced by precipitation in summer.

## Discussion

Species distribution models estimate the relationship between species records at sites and the environmental and/or spatial characteristics of those sites. MaxEnt model uses presence-only data. These data are a valuable resource and potentially can be used to model the same ecological relationships as with presence-absence data, provided that biases can be dealt with and except for the non-identifiability of prevalence [43].

The AUC value in this study was 0.869, 0.939 and 0.921 for *An. superpictus* (*s.l*.), *An. maculipennis* (*s.l*.) and *An. sacharovi*, respectively. Previous studies on MaxEnt model considered predicting AUC > 0.75 as good value for suitable niche for these species [42, 46]. This means that MaxEnt prediction in our study is very good. A recent study in Iran reported AUC values of 0.943, 0.974 and 0.956 for *Anopheles stephensi*, *An. culicifacies* (*s.l*.) and *An. fluviatilis* (*s.l*.), respectively [6]. In other countries AUC values were reported between 0.77 and 0.99 for different *Anopheles* species [47–51]. This value is affected by the ecology of the different species, number of training points for model and the variables used for modeling. However, some researchers believe AUC is a misleading measure of the performance of predictive distribution models [52].

According to the results of the Jackknife test, precipitation had the main role in modeling all three species (Fig. 4). Bio12 (annual precipitation) was the most important variable for predicting *An. superpictus* (*s.l*.) and *An. maculipennis* (*s.l*.), and Bio17 (precipitation of the driest quarter) was the most important variables for *An. sacharovi*. It is clear that increasing precipitation will increase the availability of larval habitats and also relative humidity. Therefore, mosquitoes will have more places to lay their eggs, increasing the population. Also, the longevity of adults will increase in higher relative humidity of the environment. Previous studies have shown that in southern parts of Iran with warmer weather with low precipitation, temperature was a significantly effective variable in predicting the ecological niches of malaria vectors [6]. The difference between *An. superpictus* (*s.l*.) and the reported species may be due to their ecology. This *Anopheles* species is found to have a tendency for high altitudes with higher rainfall and lower temperatures. Comparing southern malaria vectors, other environmental variables have been reported to be important factors in modeling some *Anopheles* species in other countries. Slope and land cover was relevant for *An. bellator* and *An. marajoara* in Brazil [48], altitude, stream vegetation and soil were relevant for *An. gambiae* and *An. sergentii* in Saudi Arabia [47], whereas altitude was crucial for primary malaria vectors in northern South America [46] and for *Anopheles albimanus* in Mesoamerica and the Caribbean Basin [50]. In our study, the most important environmental variable in the model for *An. superpictus* (*s.l*.) was NDVI, which was followed by altitude; both had lower importance rather than precipitation.

Based on our study, *An. superpictus* (*s.l*.) can be found with > 60% probability in the areas with annual mean temperature of 8.9–27.6 °C and mean annual rainfall of 423.55 mm. Study on this species in Europe showed it is distributed in areas where the range of temperature was between 14.9–23.7 °C and the mean rainfall was reported to be 165 mm [53]. Study on the effect of different larval rearing temperatures on the biology of *An. superpictus* (*s.l*.) under the laboratory condition showed highest survival rate at 27 °C, while all larvae in 15 °C cohort died before reaching the third-instar stage [54]. This means that although 89% of eggs hatched at 15 °C, this species needs more environmentally suitable temperatures to complete its life-cycle successfully. Although increasing temperature from 27 to 35°C resulted in shorter larvae-adult development, from 15 to 10.82 days, higher temperatures reduced survival rate of adults/larvae from 70 to 6% [54]. For *An. maculipennis* (*s.l*.), the life-cycle from egg to adult ranged from 45 days in 13.9 °C to 14 days in 24.8 °C. These values were > 60 days in 15 °C and 11.5 days in 25 °C for *An. sacharovi* [55]. Considering land cover, *An. superpictus* (*s.l*.) seems to be more distributed in Marshy areas, irrigated cropland and grassland in Europe [53] with plants, clear and flowing water breeding sites [9]. Irrigated croplands, rice fields and river edges are the main artificial and natural breeding sites for this species in Iran, therefore, it is expected that more densities of this *Anopheles* in areas having such land covers [2, 28, 56, 57]. This is consistent with findings from other studies in Europe [53, 55], while in Tajikistan this species preferred small bodies of water with rocky bottoms in riverbeds as well as on rice fields [11]. *Anopheles maculipennis* (*s.l*.) also prefers rice fields and river bed/margins in Iran [16, 23–33]. This was also in accordance with earlier studies found in Europe [55].

With regard to altitudinal distribution, and according to the location of the occurrence records, we found *An. superpictus* (*s.l*.) to within the range between 0–2716 m above sea level in Iran. The maximum recorded elevation for this species in Tajikistan was 2800 m [12]. This value was calculated as 3483 m and 2113 m for *An. maculipennis* (*s.l*.) and *An. sacharovi*, respectively, in Iran. *Anopheles sacharovi* was collected between 353–1126 m above sea level in the Sanliurfa province of Turkey [58]. These altitudes could be considered environmental suitable areas for studies of malaria vectors. However, due to global warming it is unlikely to find these species in higher altitude in future studies.

Climate change and global warming, as well as changes in land use such as dam construction, urban development and agricultural projects, have large influence on the distribution of various species of plants and animals. Therefore, having a database for potential vectors such as mosquitoes is very important. It will greatly contribute to the study of climate change and environmental effects on future distribution of these insects. Using different scenarios of climate change and existing data in the databases, it is possible to predict the temporal and spatial distribution of different mosquito species in Iran. In addition, it will enable us determine information gaps and research need in the field of Culicidae in Iran. This approach will guarantee success of the national malaria elimination program, especially considering the climatic change and adaptation. Therefore, modeling spatial and temporal distribution of all malaria vectors is a very important and essential step for successful vector control and elimination program.

The lack of accurate registration of geographical coordinates of collection site(s), a main challenge in the entomological studies, was quite obvious in this survey. Knowing the exact coordinates of the collection sites for each mosquito species will contribute to the use of mathematical models to analyze field results. It is important to note that we have used occurrence data from 1970 to 2016 and no temporal covariates. Although bioclimatic data were an average of 1950–2000, available in worldClim dataset, the values of the co-variates may have been very different at the species occurrence sites in 1970, and this is not accounted in our analysis.

## Conclusions

Malaria still remains an important mosquito-borne disease in Iran, although indigenous cases have been limited to some foci in southeastern region. Because *An. superpictus* (*s.l*.), *An. maculipennis* (*s.l*.) and *An. sacharovi*, the main malaria vectors, are distributed more or less in different areas of the country, there is transmission risk from imported cases, even in cleared-up foci. Global warming is changing the areas at risk of malaria transmission globally and in Iran it seems northern areas will more prone to malaria transmission, where the studied species exists. Planning vector control strategies is highly dependent on good understanding of the vector ecology, as well as preparing risk maps for their environmental suitability. It is also dependent on monitoring of insecticide resistance in regular intervals. Finally, environmental variables have direct effects on the temporal and spatial distribution of mosquitoes and their ability for malaria transmission. Therefore, results of this study will be beneficial for decision makers to plan and apply proper vector control measures at the right time and place for malaria vector control in Iran.

## Additional file

Additional file 1: Coordinates for collection sites. (DOCX 17 kb)

## Abbreviations

A: Arid
AA: Absolutly arid
AUC: Area under curve
CSP: Circumsporozoite proteins
DEM: Digital elevation model
EMRO: Eastern Mediterranean region
HSA: Highly semi-arid
IRS: Indoor residual spraying
ITNs: Insecticide-treated nets
MaxEnt: Maximum entropy
MSA: Moderate semi-arid
NDVI: Normalized difference vegetation index
PCR: Polymerase chain reaction
ROC: Receiver operator characteristic
SSA: Slightly semi-arid
SID: Scientific information database
SW: Semi-wet
W: Wet
