# Supplementary material for: Predicting environmentally suitable areas for Anopheles superpictus Grassi (s.l.), Anopheles maculipennis Meigen (s.l.) and Anopheles sacharovi Favre (Diptera: Culicidae) in Iran
Source: Parasit Vectors. 2018 Jul 3;11:382. doi: 10.1186/s13071-018-2973-7 (PMC6029181; doi:10.1186/s13071-018-2973-7)
Supplement: Supplementary file 1 — Coordinates for collection sites. (DOCX 17 kb) [file 13071_2018_2973_MOESM1_ESM.docx]

**Additional file 1.** Coordinates for collection sites.

***An. superpictus* (*s.l*.) collection sites: Longitude- Latitude**

44.751- 39.6516; 44.9037- 39.5416; 45.41147- 36.16121; 45.53529- 36.15573; 45.5754- 35.9752; 45.6414- 33.8799; 45.6539- 33.8838; 45.67567- 36.39592; 45.70663- 36.7618; 45.7569- 35.07501; 45.9298- 34.4441; 45.9475- 34.4974; 45.9492- 34.9676; 45.9558- 34.585; 45.9682- 34.335; 45.9797- 36.1391; 45.9844- 34.6025; 45.9848- 34.4052; 45.9854- 34.4042; 45.9885- 34.0123; 46.0088- 36.3775; 46.017- 35.062; 46.0252- 33.7048; 46.0989- 35.5407; 46.1468- 33.7188; 46.1507- 35.3619; 46.1523- 34.5761; 46.1535- 33.9113; 46.1818- 35.4652; 46.2023- 35.1605; 46.2262- 34.9955; 46.2276- 33.9572; 46.2287- 36.2286; 46.2406- 35.1204; 46.2519- 34.0829; 46.2633- 34.6156; 46.2951- 33.8211; 46.2963- 35.2418; 46.3376- 33.7905; 46.3532- 35.3031; 46.3596- 34.8557; 46.3616- 35.8379; 46.3641- 33.6104; 46.3734- 34.4629; 46.3829- 34.7822; 46.3975- 35.2738; 46.4039- 35.2627; 46.4251- 36.2091; 46.4862- 35.2751; 46.5259- 35.2881; 46.5407- 33.2536; 46.5467- 34.811; 46.5615- 33.7706; 46.5616- 35.1463; 46.5841- 36.2916; 46.5893- 33.3632; 46.6037- 33.7358; 46.6188- 34.4538; 46.6238- 35.0049; 46.6721- 33.7325; 46.6979- 33.6921; 46.7404- 33.4628; 46.7489- 33.6072; 46.7633- 35.1725; 46.7862- 33.6478; 46.7976- 33.6749; 46.8063- 35.1793; 46.8203- 33.544; 46.8292- 35.7682; 46.8486- 35.13008; 46.84866- 35.13008; 46.8653- 34.8269; 46.9225- 33.6481; 46.9309- 33.3571; 46.9315- 33.6759; 46.9807- 34.826; 46.9828- 34.9879; 46.9954- 33.6013; 47.0406- 33.3063; 47.05306- 35.79204; 47.0614- 35.3274; 47.0928- 35.8736; 47.1889- 34.2848; 47.1976- 39.3016; 47.3526- 36.0008; 47.4177- 33.1789; 47.4844- 35.2437; 47.4912- 35.2461; 47.5093- 38.4921; 47.5373- 39.0518; 47.5416- 39.2078; 47.5486- 39.1695; 47.5535- 33.1016; 47.59159- 35.39613; 47.5935- 38.4601; 47.6155- 34.2646; 47.6238- 36.1654; 47.63136- 36.82631; 47.7008- 32.2577; 47.7414- 36.6688; 47.7657- 33.2639; 47.7704- 39.6066; 47.7839- 35.6023; 47.7994- 32.9672; 47.806- 32.9786; 47.8063- 33.2743; 47.8395- 34.5059; 47.8557- 36.00559; 47.8933- 39.6558; 47.9135- 39.1597; 47.9139- 34.9772; 47.9465- 32.8947; 47.9473- 33.3609; 47.953- 39.0712; 47.95306- 36.35604; 47.9563- 33.3735; 48.0547- 33.4782; 48.1189- 33.1636; 48.1438- 33.8509; 48.2754- 33.4716; 48.2916- 39.0498; 48.3377- 34.9276; 48.4097- 33.6321; 48.4478- 31.97397; 48.4981- 33.7009; 48.6161- 34.678; 48.62779- 31.76994; 48.67165- 35.88291; 48.74712- 32.43122; 48.7741- 34.1485; 48.7827- 36.51196; 48.85181- 36.91698; 48.86817- 36.91707; 48.8871- 33.2121; 48.9277- 33.0471; 48.976- 33.0463; 49.0315- 30.73427; 49.04207- 36.85085; 49.1338- 33.1709; 49.17599- 30.72919; 49.1901- 33.1814; 49.1931- 33.1806; 49.1935- 33.1867; 49.2285- 30.75905; 49.25828- 31.83238; 49.28345- 30.80226; 49.2837- 33.1973; 49.3674- 33.1192; 49.4654- 32.8958; 49.5491- 31.2418; 49.5631- 33.4126; 49.5817- 32.827; 49.5875- 32.8121; 49.593- 33.0495; 49.5953- 31.9448; 49.5987- 33.4011; 49.6007- 32.8378; 49.60789- 31.93135; 49.608- 33.0826; 49.629- 33.4089; 49.6376- 33.0894; 49.6476- 33.027; 49.69762- 32.05543; 49.7- 33.056; 49.70713- 31.53747; 49.72146- 31.82443; 49.7216- 33.1769; 49.73417- 30.57235; 49.7358- 31.89156; 49.7378- 33.115; 49.76266- 30.97301; 49.7835- 33.0914; 49.78497- 30.59291; 49.79504- 31.78814; 49.81603- 32.05887; 49.81734- 32.00827; 49.82156- 32.04065; 49.82261- 31.37847; 49.8242- 32.0025; 49.82843- 32.03429; 49.8505- 30.8788; 49.8645- 33.0915; 49.87445- 32.00395; 49.8763- 32.4177; 49.8857- 31.5302; 49.89133- 30.5271; 49.8936- 33.2891; 49.904- 32.3875; 49.92224- 31.79405; 49.96174- 31.62344; 50.04793- 31.35949; 50.0702- 32.1731; 50.0911- 32.2043; 50.1026- 32.1935; 50.1059- 32.1548; 50.117- 32.1098; 50.1266- 32.2093; 50.1647- 33.419; 50.1649- 33.419; 50.1835- 32.992; 50.188- 32.911; 50.234- 32.9099; 50.244- 32.9108; 50.33151- 31.53013; 50.357- 32.867; 50.364- 32.867; 50.5163- 32.11; 50.51694- 30.53711; 50.526- 30.42449; 50.5428- 32.3024; 50.545- 32.876; 50.9162- 34.3386; 50.9888- 34.3423; 51.0109- 31.1483; 51.2595- 28.8884; 51.358- 29.5963; 51.3682- 28.6515; 51.3853- 35.6131; 51.3959- 35.6464; 51.416- 31.4139; 51.487- 32.364; 51.5338- 32.618; 51.5415- 29.7584; 51.54319- 32.61594; 51.56785- 30.6426; 51.57456- 30.60397; 51.61401- 30.61759; 51.6144- 35.5199; 51.6416- 29.8039; 51.6463- 35.3579; 51.7962- 29.3841; 51.8136- 32.56; 51.9221- 33.505; 52.3159- 28.5965; 53.39232- 36.56317; 54.2235- 27.1272; 54.226- 27.112; 54.2339- 31.9835; 54.2753- 31.9401; 55.23- 37.53; 55.282- 27.8462; 55.45- 37.35; 55.78- 37.57; 56.34574- 27.76212; 56.346- 38.1735; 56.4371- 38.1806; 56.62222- 36.72155; 56.7488- 33.7738; 56.8687- 37.512; 56.8914- 33.8276; 56.9201- 37.8039; 56.9849- 27.1349; 57.0347- 37.664; 57.04- 37.6349; 57.05398- 26.81899; 57.0818- 37.6957; 57.1256- 37.6536; 57.22185- 27.74205; 57.22401- 33.89095; 57.247- 37.3567; 57.2602- 28.0456; 57.3532- 37.4442; 57.4295- 26.8407; 57.4415- 37.4774; 57.4482- 27.5474; 57.4487- 27.605; 57.5233- 26.416; 57.65- 26.4166; 57.6538- 27.6371; 57.682- 28.905; 57.6833- 26.683; 57.7798- 37.44; 57.7833- 26.4833; 57.88739- 26.54361; 58.1087- 27.6723; 58.1692- 36.7642; 58.7283- 33.9603; 59.00511- 36.73247; 59.04861- 35.31453; 59.5171- 33.8317; 59.7465- 33.5235; 59.7548- 33.4201; 60.08518- 26.81436; 60.243- 33.3212; 60.7542- 26.2234; 60.77503- 27.33384; 61.13167- 35.73142; 61.21259- 26.68333; 61.23033- 26.60193; 61.40879- 26.22371; 61.42364- 26.28813; 61.68705- 26.60831; 61.77419- 26.14883; 62.19553- 26.62571; 62.52757- 26.72734

***An. maculipennis* (*s.l*.) collection sites: Longitude- Latitude**

44.4272- 39.34076; 44.4326- 39.3171; 44.43452- 39.44608; 44.4366- 39.40885; 44.4368- 39.3346; 44.65336- 39.26423; 44.65938- 37.73059; 44.6609- 39.7103; 44.66162- 37.71433; 44.68755- 37.7233; 44.69006- 39.28531; 44.7426- 39.6612; 44.751- 39.6516; 44.7855- 39.6128; 44.7964- 37.5665; 44.8325- 39.5979; 44.8336- 37.4878; 44.8514- 37.4453; 44.862- 39.60072; 44.8851- 39.5783; 44.89946- 39.3407; 44.9037- 39.5416; 44.9575- 39.4255; 44.98486- 37.65677; 44.98625- 37.4951; 44.9926- 39.2288; 45.00348- 37.55721; 45.05465- 39.36545; 45.36436- 36.95784; 45.41147- 36.16121; 45.5283- 37.00381; 45.53529- 36.15573; 45.5754- 35.9752; 45.67567- 36.39592; 45.70663- 36.7618; 45.7226- 37.766; 45.73441- 36.81477; 45.9797- 36.1391; 46.031- 35.6565; 46.0989- 35.5407; 46.2287- 36.2286; 46.3108- 35.4245; 46.3616- 35.8379; 46.3981- 36.6139; 46.4039- 35.2627; 46.6238- 35.0049; 46.8063- 35.1793; 46.8292- 35.7682; 47.1889- 34.2848; 47.1976- 39.3016; 47.3526- 36.0008; 47.4608- 38.3074; 47.471- 38.4604; 47.4844- 35.2437; 47.5- 38.483; 47.5093- 38.4921; 47.5296- 38.34151; 47.5321- 38.3434; 47.5373- 39.0518; 47.5416- 39.2078; 47.5486- 39.1695; 47.5921- 38.5123; 47.6206- 35.5157; 47.6238- 36.1654; 47.63136- 36.82631; 47.6348- 38.3559; 47.7196- 38.2975; 47.7414- 36.6688; 47.7704- 39.6066; 47.7839- 35.6023; 47.8501- 39.1278; 47.8557- 36.00559; 47.8787- 39.6467; 47.953- 39.0712; 47.95306- 36.35604; 47.9621- 39.6767; 47.9917- 39.6968; 48.0594- 39.6073; 48.1438- 33.8509; 48.3377- 34.9276; 48.4756- 38.4232; 48.476- 37.681; 48.6161- 34.678; 48.67165- 35.88291; 48.7827- 36.51196; 48.85181- 36.91698; 48.86817- 36.91707; 49.04207- 36.85085; 49.1901- 33.1814; 49.28799- 37.47773; 49.3674- 33.1192; 49.4631- 37.4615; 49.4654- 32.8958; 49.5631- 33.4126; 49.5817- 32.827; 49.5875- 32.8121; 49.5987- 33.4011; 49.6007- 32.8378; 49.629- 33.4089; 49.66626- 37.34103; 49.7- 33.056; 49.7216- 33.1769; 49.7378- 33.115; 49.7708- 37.3555; 49.7835- 33.0914; 49.80605- 37.26495; 49.8645- 33.0915; 49.88585- 37.17253; 49.8936- 33.2891; 49.904- 32.3875; 49.91265- 37.38933; 49.92648- 37.13617; 49.9425- 37.4162; 49.98234- 37.07795; 49.9902- 37.22407; 50.0127- 37.14829; 50.0702- 32.1731; 50.0911- 32.2043; 50.1026- 32.1935; 50.1059- 32.1548; 50.11356- 37.06479; 50.117- 32.1098; 50.1266- 32.2093; 50.1647- 33.419; 50.1649- 33.419; 50.1762- 37.1562; 50.1835- 32.992; 50.188- 32.911; 50.225- 37.2146; 50.234- 32.9099; 50.244- 32.9108; 50.25429- 37.23143; 50.26068- 37.09938; 50.32619- 36.73751; 50.357- 32.867; 50.364- 32.867; 50.411- 33.211; 50.4121- 32.2123; 50.5163- 32.11; 50.545- 32.876; 50.55041- 36.94177; 50.6417- 36.9225; 50.70318- 36.86117; 51.0109- 31.1483; 51.416- 31.4139; 51.487- 32.364; 51.5338- 32.618; 51.54319- 32.61594; 51.8136- 32.56; 51.9221- 33.505; 52.87341- 36.36114; 53.03851- 36.09144; 53.11378- 36.4493; 53.12343- 36.41278; 53.12706- 36.42823; 53.35779- 36.57526; 53.39232- 36.56317; 53.39946- 36.58185; 54.4778- 36.7082; 54.5829- 36.7817; 55.23- 37.53; 55.2934- 37.0563; 55.4004- 37.2261; 55.45- 37.35; 55.78- 37.57; 56.9752- 38.2135; 57.02011- 37.55856; 57.0347- 37.664; 57.1256- 37.6536; 57.4415- 37.4774

***An. sacharovi* collection sites: Longitude- Latitude**

44.6609- 39.7103; 44.7426- 39.6612; 44.751- 39.6516; 44.7855- 39.6128; 44.8325- 39.5979; 44.862- 39.60072; 44.8851- 39.5783; 44.9037- 39.5416; 44.9345- 39.4565; 44.9405- 39.4481; 44.9575- 39.4255; 44.9926- 39.2288; 46.3981- 36.6139; 46.9713- 39.1434; 47.1976- 39.3016; 47.4078- 39.4412; 47.4149- 39.4369; 47.5373- 39.0518; 47.5416- 39.2078; 47.5486- 39.1695; 47.5935- 38.4601; 47.6113- 39.5361; 47.7704- 39.6066; 47.8501- 39.1278; 47.8665- 38.9165; 47.8787- 39.6467; 47.8933- 39.6558; 47.8999- 36.6542; 47.917- 39.65; 47.9202- 39.0029; 47.9621- 39.6767; 47.9631- 38.9585; 47.9917- 39.6968; 48.0131- 38.9459; 48.058- 39.6019; 48.0594- 39.6073; 48.0713- 39.5537; 48.2196- 38.5888; 48.2916- 39.0498; 48.3371- 39.3798; 49.1931- 33.1806; 49.81734- 32.00827; 49.89133- 30.5271; 49.92224- 31.79405; 49.95458- 31.79221; 50.9162- 34.3386; 51.8184- 29.9408; 55.282- 27.8462; 59.7465- 33.5235
